# Potentially inappropriate medication use in nursing homes: an observational study using the NORGEP-NH criteria

**DOI:** 10.1186/s12877-017-0608-z

**Published:** 2017-09-19

**Authors:** Gunhild Nyborg, Mette Brekke, Jørund Straand, Svein Gjelstad, Maria Romøren

**Affiliations:** Department of General Practice/Family Medicine, HELSAM, Institute of Health and Society, University of Oslo, P.o. Box 1130 Blindern, 0318 Oslo, Norway

**Keywords:** Potentially inappropriate medication use, Elderly, Nursing homes, Drug safety, Explicit criteria, NORGEP-NH, Psychotropic medications

## Abstract

**Background:**

Frail residents in the nursing home sector call for extra care in prescribing. The Norwegian General Practice Nursing Home (NORGEP-NH) list of 34 explicit criteria for potentially inappropriate medication use in nursing homes was developed explicitly for this population. The aim of this study was to employ the NORGEP-NH Criteria to study the extent of potentially inappropriate medication use among nursing home residents and explore possible associated factors.

**Methods:**

Cross-sectional observational pharmacoepidemiological study from residents in nursing homes in the county of Vestfold, Norway. Data collected 2009–11 included residents’ demographic and clinical status and all medications, regular and on demand.

**Results:**

881 patients from 30 institutions (mean 85.9 years, 68.6% female), were included. According to NORGEP-NH, 43.8% were prescribed at least one potentially inappropriate regular medication, and 9.9% regularly received three or more potentially inappropriate medications. When also including a) the NORGEP-NH Deprescribing Criteria and b) including drugs prescribed for use as needed, 92.7% of all residents received medication that needs particular surveillance according to the NORGEP-NH. 69.7% of the nursing home residents used at least one psychotropic drug regularly. Female residents received more often than males at least one potentially inappropriate regular medication (OR 1.60, p=0.007). Regarding the prescription of three or more concomitant psychotropic medications, odds ratio for females was 1.79 (p=0.03) compared to males. Residents with the best performance in activities of daily living, and residents residing in long-term wards, had higher risk of using three or more psychotropic drugs. Use of multiple psychoactive drugs increased the risk of falls in the course of an acute episode of infection or dehydration (odds ratio 1.70, p=0.009).

**Conclusions:**

Prevalence of potentially inappropriate medications in nursing homes according to the NORGEP-NH was extensive, and especially the use of multiple psychotropic drugs. The high prevalence found in this study shows that there is a need for higher awareness of medication use and side effects in the elderly population.

**Trial registration:**

Retrospectively registered. Data obtained from clinical trial NCT01023763 registered with ClinicalTrials.gov 12/01/2009.

## Background

Due to demographic changes and an intensified effort in community care of the elderly [1], residents in European nursing homes have over the past decades become increasingly frail, often with multiple active diagnoses [2]. The situation is similar in Norway, where a recent study found that the prevalence of dementia among Norwegian nursing home residents increased from 80.4% in 2004 to 84.3% in 2010–11. The average resident is incapable of walking without assistance, and also needs assistance for other activities of daily living (ADL) [3]. Meanwhile, development in the field of pharmacology has given doctors a broader palette in their effort to treat discomfort and diseases. As a result, it is now common for nursing home residents to have medication lists of substantial length. A cross sectional study in eight European countries found that almost one out of four nursing home residents were subject to excessive polypharmacy (10 or more medications), whereas polypharmacy (5–9 drugs) was observed in one out of two [4]. A US study from 2004 found polypharmacy, defined as 9 or more medications, in 40% of nursing home residents [5].

Elderly are especially prone to side-effects and drug interactions due to physiological changes like reduced kidney, cognitive and sensory function, and altered pharmacokinetics and pharmacodynamics [6]. While medication use is crucial for symptom relief and reduction of morbidity and mortality, polypharmacy is also associated with an increased risk of adverse drug reactions (ADRs) [7, 8]. However, due to dementia and other conditions, many nursing home residents have problems expressing their opinion and experience regarding medication use, which may increase the risk of ADRs being unrecognized. Consequently, it is important that nursing home physicians are aware of the risks involved in medication use, and that medication reviews and deprescribing are prioritized tasks [9, 10].

Potentially inappropriate medications (PIMs) can be defined as drugs that pose more risks than benefits to the patients [11]. Several lists of explicit and implicit criteria have been developed for the surveillance of PIM use in a general elderly population [12–14]. The US Beers criteria have been widely used and were last updated in 2015 [15]. The Beers list reflects prescribing patterns and drugs marketed in the US. The STOPP list developed in Ireland in 2008, updated in 2015 [16], has gained increased popularity in Europe. The STOPP criteria require access to clinical information, giving rise to concern that the tool may be too comprehensive for some clinical and research purposes [17]. The NORGEP Criteria were developed in Norway in 2008, intended for use in general practice and for a home-dwelling elderly population [18].

However, multi-morbidity, frailty, and the end-of-life setting, imply that the nursing home population requires especially targeted tools for medication surveillance. For many years, the Beers 1991 list of explicit criteria for inappropriate medication use in nursing homes was the only list especially developed for the nursing home setting [11]. According to these criteria, about half of all nursing home residents have been reported to be exposed to PIMs [19–21]. The use of psychotropics is substantial in the nursing home setting, and is associated with ADRs and falls [22, 23]. In some research, the variable of three or more concomitant psychotropic drugs has been used as a substitute variable for potentially inappropriate psychotropic use [24, 25].

In order to have an updated tool for assessment of medication use in nursing homes that was also suited for the Norwegian pharmaceutical market, the Norwegian General Practice – Nursing Home (NORGEP-NH) criteria for potentially inappropriate medication use especially for elderly in nursing homes were developed through a three-round Delphi consensus process in 2012 [26] (see Table 3). In addition to the “Single substance” and “Combination” criteria parallel to those found in the original NORGEP criteria, the NORGEP-NH criteria also introduced a third category – the “Deprescribing” criteria. This category contains substances that are not inappropriate per se, but that need special attention in that the need for their continued use should be reassessed frequently.

### Aims

The purpose of this study was to assess the level of potentially inappropriate medication use in elderly nursing home residents in Norway according to the NORGEP-NH criteria. We also wanted to look at factors associated with both PIM use and the use of three or more psychotropic substances concomitantly. This is the first study to explore resident characteristics and clinical factors associated with PIMs according to these criteria.

## Methods

This study was carried out in one of Norway’s 19 counties. Eligible units were all 34 nursing homes in the county (a total of 1611 beds), of which four nursing homes declined to participate. The 30 participating nursing homes had 12–124 beds (median 41), in total 1379 beds. They had one to eight departments, and either one type of wards or a combination of wards: for rehabilitation, short term and long term care, palliative care, and special departments for patients with special needs due to behavioral and psychological symptoms of dementia.

This is a cross-sectional observational study based on medication data collected during a comprehensive pragmatic cluster randomized trial assessing whether a structured training program in administrating intravenous fluids and antibiotics in nursing homes could reduce hospital transfers and ensure high quality care locally [27]. The trial followed a stepped-wedge design with nursing homes allocated to the control group before the intervention and to the intervention group after the intervention, so that patients in need of intravenous treatment were treated in the local hospital before their nursing home received the intervention, and in the nursing homes after. For a thorough description of methods for the interventional study, see [27].

The participants in our study constitute the part of the nursing home population in need of antibiotic or intravenous fluid therapy during the study period. Data were collected from nursing home residents treated with oral antibiotics from November 2009 to December 2010 (1192 cases), and residents treated with intravenous fluids and/or intravenous antibiotics either in the nursing home or in the local hospital from November 2009 to December 2011 (330 cases). Some patients were represented several times in the interventional study: 66.1% were registered once during the study period, 20% with two episodes, and the remaining with three or more episodes requiring antibiotic or intravenous treatments. For the purpose of this study, we included data from only the first treatment episode (990 individual residents). Patients hospitalized with septicemia or hospitalized for additional diagnostics or treatment were excluded in the intervention trial. Further, we excluded patients <70 years, as the NORGEP-NH criteria were developed for nursing home residents ≥70 years, leaving a total number of 914 patients. For 33 subjects (3.6% of those eligible) medication lists were not available. These were also excluded from our study, leaving a total of 881 short-term as well as permanent nursing home residents in 30 of the 34 nursing homes in the county.

In each nursing home and in each hospital department, a nurse served as primary contact for the study team. The nurses were responsible for including the patients and recording patient information in data collection forms, and photocopying the patients’ medication charts with both regular and pro re nata (PRN, as needed) drugs. For all cases, clinical data were recorded at enrollment (day 1 in the treatment course) and at predefined days during the course of the acute illness until day 30, including delirium assessed with Confusion Assessment Method (CAM) [28]. Activity of daily living (ADL) was measured by the Barthel Index [29] which was retrospectively estimated (score 1 to 20 with increasing level of ADL) by a nurse familiar with the patient as of 14 days before the disease onset, thus representing the resident’s habitual level of functioning. Falls were recorded as either “Falls with fracture or injury” within the course of 30 days into the acute episode or “Fall/Tendency of falls” as an “unspecific new symptom”. The responses for these two variables did not always overlap. For the purpose of this study, the two variables were combined into one variable, “Falls”. The variable “Death” was recorded as “Death on day x after the start of the episode”, with cut-off set to 30 days after the episode.

Dementia could not be assessed as a possible explanatory variable due to poor quality of the underlying data.

### Statistics and analyses

IBM SPSS Statistics 22® (Armonk, New York, USA) statistical software was used for the prevalence analyses. Two SPSS syntaxes were developed for the calculation of PIMs according to the NORGEP-NH, one for substances in regular use only, and one also including PRN drugs.

STATA® (College Station, Texas, USA) was used for predictor analyses in form of bivariate and multivariate regression, with odds ratio (OR) as measure of effects size and the intra-cluster correlation coefficient (ICC) as a measure of variability between clusters.

Main outcome was the prevalence of PIMs according to the NORGEP-NH criteria. We looked at each indicator, and at the sum of PIMs per person, with and without PRN drugs.

Bivariate and multivariate predictor analyses were performed using a multilevel logistic regression model stratified on the nursing home level (xtmelogit). We performed predictor analyses for PIMs in total, and also for the single criterion “concomitant use of three or more psychotropic medications”. All predictor analyses concern the use of regular medications (exclusive PRN drugs).

Variables with statistical significance in bivariate analyses and/or clinical relevance (such as death or delirium) were chosen for the final regression model. Contingency tables showed that the subdivisions of the categories made in the final model were meaningful in the sense that all categories contained an appropriate number of observations. The Akaike Information Criterion (AIC) was also employed when deciding which variables to include in our final, multivariate, mixed effects regression model.

Pearson’s r was used to check for relationship between the Barthel score and the number of drugs given on a regular basis, and between the total number of drugs given on a regular basis and the amount of PIMs.

The total number of medications was shown to have a close, approximately linear relationship to both the number of PIMs and the prescribing of 3 or more psychotropic drugs and we found a positive correlation between total number of drugs and both PIMs (Pearson’s *r* = 0.36, *p* = 0.000) and 3+ psychotropic drugs (*r* = 0.338, p = 0.000). Therefore, the total number of medications given was treated as an effect mediator and was omitted as a variable in the regression analyses in order to avoid over-adjustment bias in the estimate of OR [30].

Barthel ADL scores were categorized in tertiles.

The ICC was obtained to examine the amount of variability between the nursing homes that was not explained by the variables in the model.

We used the Chi-square test (*p* < 0.05) to check for statistical differences between patients included in and patients excluded from our study.

## Results

Overall mean age was 85.9 (range 70–102), and 604 of the 881 (68.6%) were female. 97 of the participants (11.0%) died within 30 days into the study period.

For sample characteristics, see Table 1.

**Table 1:** Sample characteristics

	Total number (%) in data set	Number with medication list (%). Included in study	Number without medication list (%). Not included in study	Mean (range) among those included in study
Participants	914 (100)	*881* (100)*	33 (100)	
Gender:				
Female	623 (68.2)	604 (68.6)	19 (57.6)	
Male	291 (31.8)	277 (31.4)	14 (42.2)	
Age (years):				85.9 (70–102)
≤ 85	401 (43.9)	382 (43.4)	19 (57.6)	
> 85	513 (56.1)	499 (56.6)	14 (42.4)	
Institutions	30	30^a^	16^a^	
No. of beds	1379			46.0^b^ (12–124)
No. of incl. Cases		881 (100)		29.4^b^ (3–170)
No. of NH doctors	57			1.9^b^ (1–6)

^a^No. of institutions represented among patients with/without medication lists. ^b^Per institution

* significant at *p*<0.05

The average number of medications given to each patient on a regular basis was 6.7 (range 0–19). When including PRN medications, the average number of medications for each patient was 9.7 (range 1–25).

For those without medications lists, age range was 74–97, mean age 84.1 years. The Chi-square test revealed no statistical differences between included and excluded patients regarding age and gender.

The NORGEP-NH Criteria and the prevalence of PIMs are given in Table 2.

**Table 2:** Prevalence of potentially inappropriate medication use in nursing home residents ≥70 years according to NORGEP-NH

NORGEP-NH^a^ List of Explicit Criteria	Freq., regular med. Only, in %	Freq., incl. PRN^b^ medication, in %
A: Single Substance Criteria. The following should be avoided for regular use whenever possible:		
1. Combination analgesic with codeine/paracetamol	0.8	6.8
2. Tricyclic antidepressants (TCAs) for depression	0.9	0.9
3. NSAIDs	1.1	7.7
4. First generation antihistamines	4.5	6.0
5. Diazepam	1.4	10.7
6. Oxazepam: Dosage >30 mg/day	0.8	N/A
7. Zopiclone: Dosage >5 mg/day	14.1	N/A
8. Nitrazepam	2.8	3.6
9. Flunitrazepam	0.3	0.3
10. Chlometiazole	1.2	8.7
11. Regular use of hypnotics	30.9	N/A
B: Combination Criteria. The following drug combinations should be avoided whenever possible:		
12. Warfarin + NSAIDs	0.0	0.5
13. Warfarin + SSRI/SNRI	1.6	1.6
14. Warfarin + ciprofloxacin/ofloxacin/ erythromycin/ clarithromycin	0.3	0.5
15. NSAIDs/coxibs + ACE-inhibitors/AT2-antagonists	0.2	1.1
16. NSAIDs/coxibs + diuretics	0.6	3.9
17. NSAIDs/coxibs + glucocorticoids	0.0	0.0
18. NSAIDs/coxibs + SSRI/SNRIs	0.2	2.0
19. ACE-inhibitors/AT2-antagonists + potassium or potassium-sparing diuretics	1.9	1.9
20. Beta blocking agents + cardioselective calcium antagonists	0.0	0.1
21. Erythromycin/clarithromycin + statins	0.1	0.1
22. Bisphosphonate + proton pump inhibitors	1.6	1.7
23. Concomitant use of three or more psychotropic drugs	14.5	41.5
24. Tramadol + SSRIs	1.4	6.1
25. Metoprolol + paroxetine/fluoxetine/bupropion	0.0	0.0
26. Metformin + ACE-inhibitors/AT2-antagonists + diuretics	1.0	1.0
C: Deprescribing criteria. Need for continued use should be reassessed:		
27. Anti-psychotics	10.3	14.2
28. Anti-depressants	35.3	35.5
29. Urologic spasmolytics	0.7	0.7
30. Anticholinesterase inhibitors	5.9	6.0
31. Drugs that lower blood pressure^c^	62.5	65.2
32. Bisphosphonates	5.4	5.6
33. Statins	12.1	12.1
34. General use of preventive medication	N/A^d^	N/A^d^

^a^The Norwegian General Practice criteria for assessing potentially inappropriate prescriptions to elderly patients in Nursing Homes. ^b^Pro re nata, drugs given as needed. ^c^Incl. in the figures: All drugs that have the lowering of blood pressure as primary outcome (i.e. hypertensives). Excl. drugs with lower blood pressure as side effect, wanted or unwanted. ^d^Criterion 34 on the NORGEP-NH list, “General use of preventive medication”, was not assessed in this paper, as information was lacking on whether medication was given for the purpose of treatment or prevention. Abbreviations: NSAIDs: Non-steroid anti-inflammatory drugs. SSRIs: Selective serotonin reuptake inhibitors. SRNIs: Selective norepinephrine reuptake inhibitors. Coxibs: Cyclooxygenase-2-selective inhibitors. ACE-inhibitors: Angiotensin-converting enzyme inhibitors. AT2-antagonists: Angiotensin II receptor antagonists

Over 10% of the residents used antipsychotics, 30.9% used hypnotics, and 35.3% used antidepressants on a regular basis. Three or more psychotropic drugs were used concomitantly by 14.5% of residents on a regular basis. When including the drugs on the PRN medication list, 41.5% used three or more, and one out of ten (10.5%) used five or more psychotropic drugs concomitantly (Table 3). 85.2% received one or more psychotropic substances, regularly or on demand.

**Table 3:** Prevalence of PIMs per person according to the NORGEP-NH criteria

No. of PIMs per person	NORGEP-NH A^1^+B^2^ + C^3^, prevalence (%),		NORGEP-NH A^1^+B^2^, prevalence (%)		No. of psychotropic drugs per person, prevalence (%)	
	Excl. drugs on demand	Incl. drugs on demand	Excl. drugs on demand	Incl. drugs on demand	Excl. drugs on demand	Incl. drugs on demand
0	108 (12.3)	64 (7.3)	495 (56.2)	265 (30.1)	267 (30.3)	130 (14.8)
1	253 (28.7)	163 (18.5)	163 (18.5)	185 (21.0)	290 (32.9)	182 (20.7)
2	211 (24.0)	161 (18.3)	136 (15.4)	181 (20.5)	196 (22.2)	203 (23.0)
3	142 (16.1)	151 (17.1)	63 (7.2)	138 (15.7)	86 (9.8)	160 (18.2)
4	80 (9.1)	130 (14.8)	20 (2.3)	72 (8.2)	37 (4.2)	113 (12.8)
5	52 (5.9)	104 (11.8)	3 (0.3)	22 (2.5)	4 (0.5)	53 (6.0)
6	26 (3.0)	57 (6.5)	0	13 (1.5)	1 (0.1)	29 (3.3)
7	5 (0.6)	29 (3.3)	1 (0.1)	2 (0.2)	0	8 (0.9)
8	3 (0.3)	13 (1.5)	0	3 (0.3)	0	2 (0.2)
9	0	3 (0.3)	0	0	0	1 (0.1)
10	1 (0.1)	5 (0.6)	0	0	0	0
11	0	1 (0.1)	0	0	0	0
SUM	881 (100%)	881 (100%)	881 (100%)	881 (100%)	881 (100%)	881 (100%)

^1^A: Single substance criteria ^2^B: Combination criteria ^3^C: Deprescribing criteria

Number of people affected, with percentages

Of the nursing home residents in this study, 43.8% had at least one PIM, according to the NORGEP-NH criteria parts A and B (Table 3). When including PRN drugs, the percentage of residents affected by at least one PIM rose to 69.9%. One in ten (9.9%) was given three or more PIMs concurrently on a daily basis. Only 7.2% of residents did not receive any medication according to the NORGEP-NH Single substance, Combination, and Deprescribing criteria.

### Factors associated with potentially inappropriate medication

We ran analyses for both PIMs in total, and for the specific criterion “Concomitant use of three or more psychotropic drugs”, in the regression models. Bivariate and multivariate mixed effects regression results with odds ratios, confidence intervals and *p*-values for all residents are shown in Table 4. The results commented below are all from multivariate analyses.

**Table 4:** Factors associated with potentially inappropriate medications, and 3+ concomitant psychotropic medications according to NORGEP-NH Single Substance and Combination Criteria^a^

		PIMs according to NORGEP-NH Criteria 1–26				3+ concomitant psychotropic drugs			
Variable	No. of residents n (valid cases, %)	Bivariate analyses OR (95% C.I.)	*p*	Multivariate analyses OR (95% C.I.)	*p*	Bivariate analyses OR (95% C.I.)	*p*	Multivariate analyses OR (95% C.I.)	*p*
Age^b^ *n* = 881	881	0.99 (0.97–1.01)	0.34	0.98 (0.96–1.01)	0.17	0.99 (0.96–1.02)	0.59	0.98 (0.95–1.01)	0.18
Gender: n = 881 Male	277 (31.4)	1 (Ref)		1 (Ref)		1 (Ref)		1 (Ref)	
Female	604 (68.6)	1.38 (1.02–1.86)	0.03*	1.60 (1.14–2.24)	<0.01*	1.72 (1.09–2.71)	0.02*	1.79 (1.06–3.01)	0.03*
Barthel^c^ *n* = 807 0–5	314 (35.6)	1 (Ref)		1 (Ref)		1 (Ref)		1 (Ref)	
6–10	259 (29.4)	1.28 (0.91–1.80)	0.16	1.36 (0.95–1.94)	0.09	1.26 (0.75–2.10)	0.38	1.41 (0.83–2.42)	0.21
11–20	234 (26.6)	1.30 (0.91–1.85)	0.15	1.32 (0.91–1.92)	0.15	1.83 (1.11–3.04)	0.02*	2.16 (1.25–3.73)	0.01*
Falls: *n* = 830 No	663 (75.3)	1 (Ref)		1 (Ref)		1 (Ref)		1 (Ref)	
Yes	167 (19.0)	1.09 (0.77–1.54)	0.47	1.10 (0.76–1.36)	0.60	1.84 (1.16–2.91)	<0.01*	1.70 (1.03–2.80)	0.04*
Delirium^d^: *n* = 877 No	807 (91.6)	1 (Ref)		1 (Ref)		1 (Ref)		1 (Ref)	
Yes	70 (7.9)	0.93 (0.56–1.55)	0.78	0.95 (0.51–1.76)	0.88	1.22 (0.62–2.42)	0.57	1.21 (0.52–2.83)	0.66
Death^e^: *n* = 897 No	782 (88.8)	1 (Ref)		1 (Ref)		1 (Ref)		1 (Ref)	
Yes	97 (11.0)	0.75 (0.48–1.17)	0.21	0.80 (0.47–1.36)	0.41	0.97 (0.52–1.78)	0.91	1.67 (0.82–3.38)	0.16
Ward: *n* = 837 Rehabilitation	58 (6.6)	1 (Ref)		1 (Ref)		1 (Ref)		1 (Ref)	
Short-time	158 (17.9)	0.74 (0.30–1.39)	0.44	0.87 (0.39–1.94)	0.73	2.30 (0.54–9.87)	0.26	2.75 (0.60–12.60)	0.19
Long-time	364 (41.3)	1.25 (0.52–2.35)	0.57	1.61 (0.72–3.62)	0.25	5.08 (1.27–20.23)	0.02*	7.48 (1.74–32.08)	<0.01*
Short−/longtime	100 (11.4)	1.56 (0.59–3.17)	0.31	2.12 (0.86–5.26)	0.10	5.92 (1.34–26.27)	0.02*	7.20 (1.51–34.36)	0.01*
Dementia	114 (12.9)	1.11 (0.41–2.16)	0.80	1.19 (0.50–2.83)	0.70	7.24 (1.68–31.27)	<0.01*	7.70 (1.68–35.32)	<0.01*
Palliative	46 (5.2)	1.51 (0.56–3.61)	0.38	1.98 (0.73–5.40)	0.18	1.88 (0.30–11.71)	0.50	2.04 (0.30–13.99)	0.47

^a^Bivariate and multivariate mixed effects regression analyses stratified on the nursing home level. ^b^Continuous variable. ^c^No. of residents in each tertile. ^d^In the course of the infection. ^e^Within 30 days of study inclusion

*significant at *p*<0.05

Regression model including all nursing home residents

Residents’ age was not a significant predictor of PIMs or 3+ psychotropic drugs when looking at residents in all wards. However, in an alternative regression model only addressing residents in long-term and dementia wards, the odds for receiving 3+ psychotropic drugs significantly decreased with increasing age (OR 0.95, 95% C.I. 0.92–0.99, *p* = 0.02) (not shown in table). We did not find clear non-linear associations when age was tested as categorical variable in various age groups.

Female residents had higher odds of receiving PIMs than male residents (OR 1.60, *p* = 0.007). When analysing for 3+ psychotropic medications the gender difference increased (OR 1.79, *p* = 0.03). When analysing gender difference employing the alternative regression model only including residents living in long-term wards (comprised of long-time and dementia wards), OR for PIMs for female residents vs. male residents was 1.63 (95% C.I. 1.01–2.66, *p* = 0.04). However, for 3+ psychotropic drugs the odds for females increased to 2.91 (95% C.I. 1.36–6.23, *p* = 0.006) when only including residents in long-term facilities (not shown in table).

The odds of receiving PIMs were higher for the group with the highest ADL score. The odds of receiving 3+ psychotropic drugs was even higher for those with the highest ADL score, OR = 2.16 (p = 0.006) for the best functioning tertile compared to the group with the lowest ADL score. For only residents in long-term wards, this tendency was even stronger: The best functioning elderly in long-term care had an OR of 3.07 (95% C. I. 1.5–6.3, *p* = 0.002, not shown in table) of receiving 3+ psychotropic drugs compared to the group with the lowest ADL score. They also had an OR of 2.64 (95% C. I. 1.56–4.46, *p* < 0.001) of receiving PIMs compared to the lowest functioning group. When checking for relationship between the ADL score and the number of drugs given on a regular basis we did not find such correlation.

There were no significant differences between the different types of wards regarding total numbers of PIMs. However, when looking at the prescribing of 3+ psychotropic drugs, the odds were significantly higher for residents in long-term wards, dementia wards, and in wards with combined long- and short-term beds, as compared to residents in short-term wards, rehabilitation wards and palliative wards.

In the multilevel model, an ICC estimate of 0.06 was obtained, hence 6% of the variability in PIMs total can be attributed to differences between nursing homes. ICC increased to 0.16 when analysing residents receiving 3+ psychotropic drugs. When looking at only residents in long-term facilities, the difference between the nursing homes measured by the ICC increased to 0.14 for PIMs and 0.26 for 3+ psychotropic drugs.

Residents receiving 3+ psychotropic drugs had higher odds of falls in the course of the infection or dehydration episode, with or without fracture (OR 1.70, *p* = 0.04). We found no significant results on either PIMs or psychotropic drugs regarding delirium or death following in the course of the infection.

We tested a regression model where the outcome variable was medications lowering blood pressure, to see if residents using these substances were more prone to falls than other residents, but we did not find any such relationship in these data.

## Discussion

### Summary of results and comparison to previous literature

The prevalence of PIMs according to the NORGEP-NH Single Substance and/or Combination Criteria was 43.8% excluding, 69.9% including PRN medication. The use of psychotropic medications was extensive. Females were at higher risk of receiving PIMs and multiple psychotropic drugs, especially when in long-term facilities. Those with good ADL-functioning were at higher risk of receiving multiple psychotropic drugs. The use of multiple psychotropic drugs increased the risk of falls in the course of an infection or dehydration episode.

The use of first generation antihistamines is not included in the criterion regarding “Concomitant use of three or more psychotropic drugs”. Among the 4.5% using first generation antihistamines, 45% also used three or more psychotropics. Thus, these residents had yet an additional burden regarding risk of falls and over-sedation [31].

Clomethiazole is still in use for neuropsychiatric symptoms like agitation and aggression in Norway, as one of few countries, in spite of a considerably higher mortality than other substances used on same indications, and poor documentation on safety [32]. Almost one in ten (8.7%) had clomethiazole listed as a regular or PRN drug.

When analyzing “The use of TCA against depression”, we did not have information on whether the resident was using TCA to treat depression or as adjuvant treatment of chronic pain. The prevalence for this indicator may therefore be higher than the number actually using TCAs for depression. The basis for this indicator is that the anticholinergic effects of TCAs are potentially harmful for the frail elderly [33, 34]. However, Coupland et al. found newer antidepressants to be associated with an even higher risk of falls than TCAs [35]. The efficacy of antidepressants in this population is not clearly established [35–37]. There is a need for further research into effects and side effects of antidepressant drugs in this population.

The total PIM prevalence of 43.8% in our study is in accordance with a nursing home study that according to the STOPP criteria reported a prevalence rate of 46.2% [21], one study employing the Beers criteria that found a prevalence rate of 46.5% [20], and another 50%, the latter looking at residents with a minimum of three months’ stay [19]. A Norwegian study reporting prevalence rates of PIMs in nursing homes based on 28 of the 36 original NORGEP criteria developed for home-dwelling elderly found a prevalence of PIM use at 31% [38]. A recent study employing the NORGEP-NH criteria found that PIMs in Norwegians nursing homes have increased over the years 1997–2011, while the average number of drugs also increased over the same time period [39].

Ruths et al. found an increase in the regular use of psychotropic drugs among Norwegian nursing home residents from 57.6% in 1997 to 70.5% in 2009 [40]. This is in accordance with our finding of 69.7%. It has been shown that deprescribing of anti-psychotic drugs in nursing home populations may improve inhabitants’ function [41]. The high level of psychotropic medication use in the nursing home population is concerning, considering the limited effects and the high probability for serious side effects. This especially applies to antipsychotics used for behavioural and psychological symptoms of dementia (BPSD) [41–46].

We found that residents living in long-term facilities had higher odds of receiving multiple psychotropic drugs than elderly in short-term wards, rehabilitation wards or palliative care units, and among these, the elderly with the best preserved level of functioning had the highest odds of receiving three or more psychotropic drugs. This seemingly counter-intuitive result was not explained by a higher rate of deprescribing in the lowest functioning group, as we found no correlation between the ADL score and the number of drugs given on a regular basis. Instead, it seemed likely that the better functioning residents receive more psychotropic drugs, at least partly, as treatment for agitation, confusion and other neuropsychiatric symptoms in dementia, assuming the prevalence of dementia is even higher in long-term facilities than in all facilities as a total. This interpretation is consistent with the results from a recent multinational European study, where the strongest correlate of antipsychotic drug use was found to be severe behavioral symptoms [47], and Lovey et al. [48] who found that the use of antipsychotics was correlated to aggressive, verbally disruptive and wandering behavior and the ability to rise from a chair. This finding also suggests that the level of ADL could act as a confounder in analyses regarding the use of multiple psychotropic drugs in studies where one cannot correct for ADL as a variable as was done here.

There was an unexplained variability between the nursing homes regarding the prescribing of multiple psychotropic substances, as shown by the larger ICC between nursing homes, suggesting some degree of individual differences in prescription practice between doctors, or different prescription tradition between the different nursing homes. This is consistent with findings of two other studies revealing large unexplained variance between nursing homes regarding prescribing of antipsychotics to residents often lacking a clear indication [49, 50].

Some studies have demonstrated increased levels of falls with increasing levels of PIMs [51, 52]. In this study, we found the risk of falls in the course of an acute infection or dehydration to increase for those who received multiple psychotropic drugs, but there was no clear such association when looking at the number of PIMs as a whole. We did not find a relationship between a tendency of falls and the number of blood pressure lowering substances. In a systematic review from 2007, Hartikainen et al. found [23] antihypertensive drugs to be weakly associated with falls, and psychotropics – mainly benzodiazepines, antidepressants, and antipsychotics – to be strongly associated with falls in the elderly.

The gender differences found in our study are consistent with results from a large national study of home-dwelling elderly in Norway conducted in 2008 [53], which found an odds ratio for females for receiving one or more PIMs of 1.60. There is still a need to explore further the reasons behind these differences.

### Strengths and limitations

This study is based on comprehensive information about both regular medications and medications given on demand. In addition, the clinical information provided gave an opportunity to study some clinical factors related to PIM use in this setting. The different statistical models yielded robust results regarding significant and non-significant outcomes.

The patients included in this study were selected based on their need of antibiotic or fluid treatment for acute infection or dehydration. This selection could imply a bias towards the more frail of the residents. However, infections are common among nursing home residents and the proportion that receive antibiotic treatment each year is substantial [54, 55]. We do not have access to the exact number of those included in this study in relation to the total number of residents in the 30 nursing homes, partly due to high turnover of residents and partly because this was beyond the scope of the intervention trial [27]. The county of Vestfold has 1379 nursing home places in total (for all 34 nursing homes and all age groups). Our selection of 881 patients implies that this study encompasses a fairly high proportion of the residents in the participating nursing homes. Importantly, the study design opened up for a chance to study how PIMs may affect the frail population of nursing home residents when they encounter acute illness.

Medication lists were recorded on day 1 of inclusion into the original interventional trial. As we based our analyses solely on the medication lists recorded at inclusion in the interventional trial, the intervention per se did not influence the results of our study.

The observational design allows us to analyse factors that are related to PIMs, and to 3+ psychotropics. However, this methodology does not allow us to say whether the one or the other factor is causing this relationship, or whether a common third variable (confounder) is the cause of the association [56].

### Implications for further research and practice

The highly prevalent use of PIMs in nursing homes found in this study shows that there is a need for intensified measures towards this problem. The topic should be prioritized both in research and in educational efforts in nursing home medicine in order to optimize patient treatment. The complexity of the task of prescribing to this population should be recognized by health administrators to ensure that prescribers are given sufficient resources.

However, although the high prevalence of psychotropic drugs and of PIMs in general shown here is of concern, it is important that elderly people are not withheld from efficient pharmacological treatment. Notably, adequate management of pain has been shown to reduce other use of psychotropic medications [57]. There may well be instances where the use of substances on lists as the NORGEP-NH may be appropriate. Explicit criteria like the NORGEP-HN and Beers’ criteria are meant to heighten the awareness of clinicians and caregivers to the use of these substances and the risk involved: “The criteria are designed to support, rather than supplant, good clinical judgment.” [58].

Studies over the past few years have demonstrated the widespread problem of potentially inappropriate prescribing in elderly throughout the world [53, 59–64]. There is a known relationship between ADRs and hospitalization and death [8, 65]. So far, there is conflicting evidence as to the link between PIMs and ADRs [66], and the effect of PIMs on mortality, morbidity and quality of life (QoL) [67–77]. Some studies find increased hospitalization and mortality rates and reduced QoL with increasing drug burden [75]. Medication reviews has been advocated as a means to reduce the prevalence of PIMs in nursing homes [78]. However, a recent systematic review and meta-analysis that looked at the effect of medication reviews on nursing home resident’s mortality or hospitalization found no clear correlation [79]. There is thus a need for more research into the impact of PIMs, and how we best are to reduce their prevalence [80, 81].

## Conclusion

This study analyzed potentially inappropriate medication use in nursing homes according to the NORGEP-NH criteria. We found a high prevalence of PIMs, and among these, the use of psychotropic drugs was especially prevalent. Females were at higher risk of receiving both PIMs and multiple psychotropic drugs concurrently. Residents in long-term wards, and residents with a better-preserved ADL, had a higher risk of receiving multiple psychotropic drugs. The use of multiple psychotropic drugs increased the risk of falls in the course of an infection or dehydration episode.

A prevalence of PIMs of this magnitude reveals a need for targeted measures.

## Abbreviations

ACE-inhibitors: angiotensin-converting enzyme inhibitors
ADR: adverse drug reaction
ARB: angiotensin receptor blockers
AT2-antagonists: angiotensin II receptor antagonists
BPSD: Behavioural and Psychological Symptoms of Dementia
Coxibs: cyclooxygenase-2-selective inhibitors
NH: nursing home
NORGEP-NH Criteria: Norwegian General Practice – Nursing Home Criteria
NSAIDs: non-steroid anti-inflammatory drugs
OR: odds ratio
PIM: potentially inappropriate medication
PRN: pro re nata (medication given on demand only)
QoL: quality of life
RCT: randomized controlled trial
SRNIs: selective norepinephrine reuptake inhibitors
SSRIs: selective serotonine reuptake inhibitors
TCAs: tricyclic antidepressants
